# Epicardial adipose tissue, metabolic disorders, and cardiovascular diseases: recent advances classified by research methodologies

**DOI:** 10.1002/mco2.413

**Published:** 2023-10-24

**Authors:** Yujie Song, Yanzhen Tan, Meng Deng, Wenju Shan, Wenying Zheng, Bing Zhang, Jun Cui, Lele Feng, Lei Shi, Miao Zhang, Yingying Liu, Yang Sun, Wei Yi

**Affiliations:** ^1^ Department of Cardiovascular Surgery Xijing Hospital The Fourth Military Medical University Xi'an China; ^2^ Department of General Medicine Xijing Hospital The Fourth Military Medical University Xi'an China

**Keywords:** cardiovascular disease, diabetes, epicardial adipose tissue, obesity

## Abstract

Epicardial adipose tissue (EAT) is located between the myocardium and visceral pericardium. The unique anatomy and physiology of the EAT determines its great potential in locally influencing adjacent tissues such as the myocardium and coronary arteries. Classified by research methodologies, this study reviews the latest research progress on the role of EAT in cardiovascular diseases (CVDs), particularly in patients with metabolic disorders. Studies based on imaging techniques demonstrated that increased EAT amount in patients with metabolic disorders is associated with higher risk of CVDs and increased mortality. Then, in‐depth profiling studies indicate that remodeled EAT may serve as a local mediator of the deleterious effects of cardiometabolic conditions and plays a crucial role in CVDs. Further, in vitro coculture studies provided preliminary evidence that the paracrine effect of remodeled EAT on adjacent cardiomyocytes can promote the occurrence and progression of CVDs. Considering the important role of EAT in CVDs, targeting EAT might be a potential strategy to reduce cardiovascular risks. Several interventions have been proved effective in reducing EAT amount. Our review provides valuable insights of the relationship between EAT, metabolic disorders, and CVDs, as well as an overview of the methodological constructs of EAT‐related studies.

## Graphical abstract

Considering the advances in research and biological technology, our understanding of EAT has undergone a continuous deepening process, from volumetric measurement to in‐depth profiling of EAT, and from correlational to cause–effect studies. EAT amount measured by imaging techniques is significantly higher in patients with metabolic disorders. An increased EAT volume is associated with a higher risk of developing metabolic syndrome, cardiovascular mortality, atrial fibrillation (AF) incidence, and reduced cardiac function. Further, in‐depth profiling studies suggest that EAT is remodeled into a more proinflammatory phenotype in patients with metabolic disorders. The levels of proinflammatory cytokines such as IL‐1, IL‐6, TNF‐α, and interferon‐gamma in EAT under conditions of metabolic disease are significantly higher than those in the control group. Additionally, the expression of scavenger receptors (SRs) and macrophage infiltration are at higher levels. Finally, coculture studies explored the paracrine effect of EAT. Studies found that the secretory profile of EAT form patients with metabolic disorders is distinct from the control group. Moreover, in vitro coculture studies, in which cardiomyocytes were cultured with secretory products of EAT biopsies from patients, have shown that secretory products of EAT from patients with metabolic disorders exhibited detrimental effects on cardiomyocytes, such as impairing the contractile function of cardiomyocytes. Furthermore, coculture of the cardiomyocytes with secretory products of EAT from AF patients resulted in changes in cardiomyocyte electrophysiology and increased cardiac fibrosis, which are related to the mechanisms underlying AF.

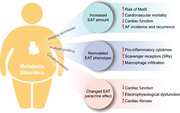

## 1 INTRODUCTION

Obesity and diabetes are among the primary risk factors for cardiovascular morbidity and mortality and are known as cardiometabolic risk factors.^1^, ^2^, ^3^, ^4^, ^5^ In individuals with obese and diabetic, a portion of adipose tissues becomes dysfunctional and significantly contributes to the chronic low‐grade inflammation in this group.^6^, ^7^ This inflammation state is known as metabolic‐associated inflammation and is associated with increased cardiovascular risk.^8^, ^9^

Notably, the properties of different fat depots can vary significantly in many aspects, including composition (white or brown), physical function (fat storage or thermogenesis), inflammation state, and secretome. Consequently, their roles in health and disease vary widely.^10^ For instance, excessive accumulation of visceral adipose tissue (VAT) has been found to be a major cardiovascular risk factor that promotes the production of proinflammatory adipokines, but subcutaneous adipose tissue (SAT) is not.^11^, ^12^, ^13^, ^14^ This highlights the importance of the location and properties of fat deposits. Epicardial adipose tissue (EAT) is a special type of VAT that is located between the myocardium and visceral pericardium. EAT has great potential in locally influencing adjacent tissues such as the myocardium and coronary arteries and may act as a local transducer of systemic inflammation in obese or diabetic patients.^15^ Thus, EAT has received extensive attention in the past decade, and there is a rapidly growing body of research exploring the association between EAT, metabolic disorders, and cardiovascular diseases (CVDs).^16^, ^17^, ^18^, ^19^, ^20^, ^21^, ^22^, ^23^, ^24^

Studies in the area of EAT can be classified into three categories according to their research methodology. The first category is studies based on imaging techniques, which accounts for the majority of EAT‐related studies. Several features of EAT, including thickness, volume, average computed tomography (CT) attenuation and radiomic features can be acquired by imaging techniques, which are valuable to study the relationship between EAT and diseases. The second category of studies is based on profiling of EAT clinical specimens using molecular biology techniques, such as polymerase chain reaction (PCR), enzyme‐linked immunosorbent assay (ELISA), and sequencing technology. This category of studies expanded our knowledge of EAT into the molecular level, which we referred here as in‐depth profiling studies. Due to the absence of EAT in rodents and lack of means to specifically intervene EAT in the human body, it is challenging to establish a causal relationship between EAT and CVDs in vivo and in randomized controlled trials (RCTs). The third category of studies, in vitro cocultural studies, provide an alternative. By incubating cell models of CVDs with secretory products generated from explants of human EAT samples, these studies have provided preliminary evidence of the causal relationship between EAT and CVDs.

**FIGURE 1:**
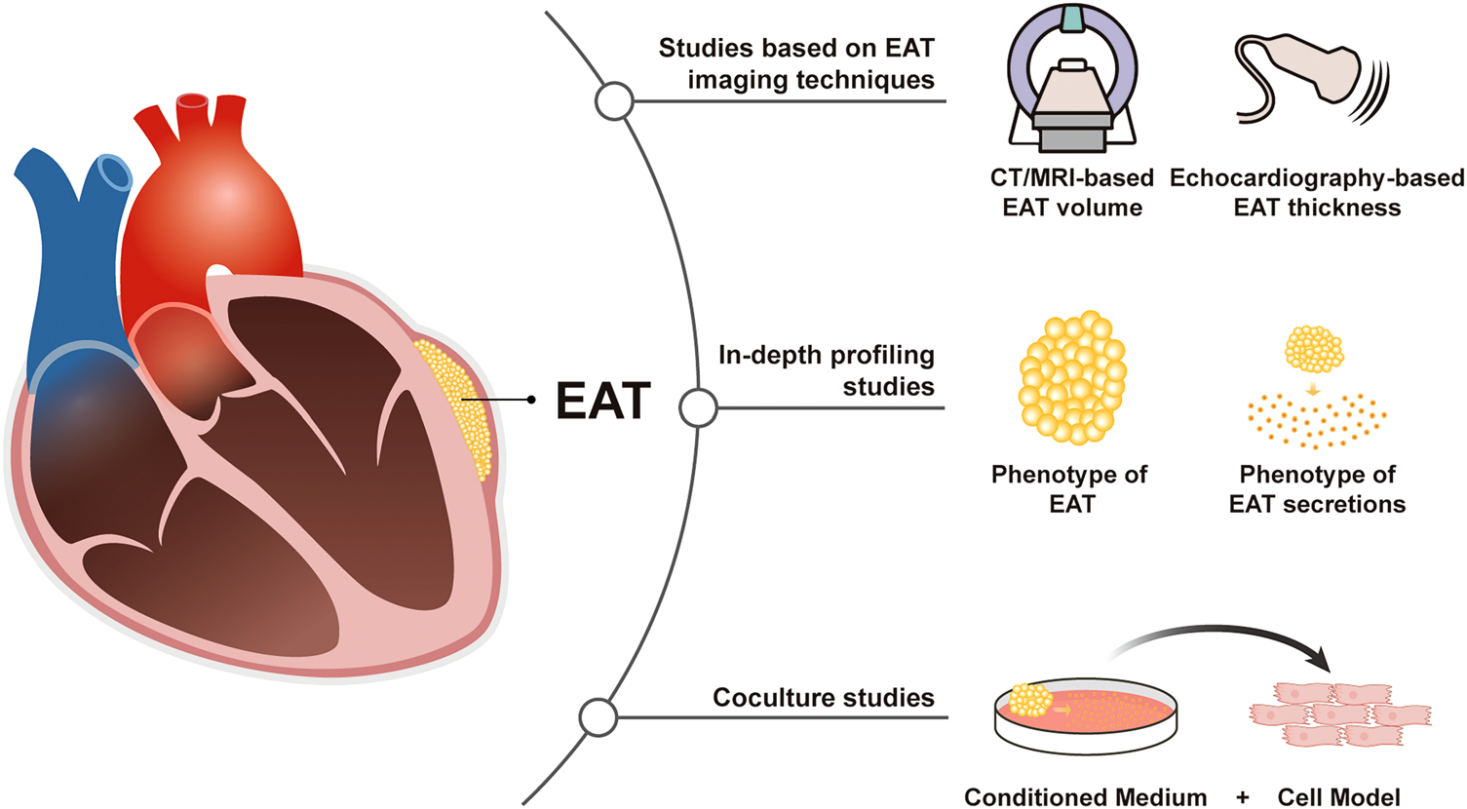
Three categories of research in the area of epicardial adipose tissue (EAT). Studies in the area of EAT can be classified into three categories according to their research methodology, namely studies based on imaging techniques, in‐depth profiling of EAT, and in vitro cocultural studies. Simultaneously, these three categories of investigations also represent the continuous deepening process of our understanding of EAT. First, studies based on imaging techniques made important contributions to demonstrate the association between EAT and cardiovascular diseases (CVDs). Second, in‐depth profiling of EAT and its secretome allowed us to gain deeper insights into the key role played by EAT in CVDs. Third, coculture studies were conducted to explore the paracrine effects of EAT on cardiomyocytes.

In this review, we first summarized the anatomic characteristics and physiological function of EAT. Then, classified by research methodologies, we reviewed the recent advances of the relationship between EAT, metabolic disorders, and CVDs. Using this unique perspective, we avoided mixing conclusions drawn from studies with distinct levels of evidence, for example, clinical and basic studies. At the same time, we were able to provide an overview of the methodological constructs of the studies in the field of EAT research, as well as summarize the continuous deepening process of our understanding of EAT, from volumetric measurement to in‐depth profiling of EAT, and from correlational studies to cause–effect studies (Figure 1). Finally, in the last part, we reviewed clinical studies exploring the possibility of targeting EAT as a therapeutic strategy.

## 2 ANATOMIC CHARACTERISTICS AND PHYSIOLOGICAL FUNCTION OF EAT

EAT is defined as adipose tissue located between the myocardium and visceral pericardium and is distinct from paracardial adipose tissue (PAT), which is located external to the parietal pericardium. From the perspective of embryo development, EAT originates from the splanchnopleuric mesoderm, while PAT originates from the primitive thoracic mesenchyme.^25^ Besides, EAT is supplied by coronary artery and shares microcirculation with the myocardium. In comparison, PAT is supplied by noncoronary sources.^26^, ^27^ Moreover, EAT is in direct contact with the underlying myocardium, with no fascia separating them, further enabling paracrine interaction. In human and large mammals, EAT covers 80% of the heart's surface and accounts for approximately 14.7% of the total heart weight.^28^, ^29^, ^30^, ^31^ However, EAT is almost absent in laboratory mice and rats, which makes it challenging to conduct EAT‐related animal experiments.^30^

Histologically, EAT is mainly composed of adipocytes, other components include nerve tissues, stromovascular, inflammatory, and immune cells. The adipocytes of EAT are smaller than those in subcutaneous and other visceral fat depots. EAT is generally considered as white adipose tissue, but also exhibits brown fat‐like or beige fat‐like features. The marker of brown adipose tissue, UCP‐1 is highly expressed in EAT.

Under physiological conditions, the proposed function of EAT includes thermogenesis, serving as a source of free fatty acids (FFAs), and mechanical functions.^29^, ^32^, ^33^ However, more studies are needed to further validate these functions. First, it is speculated that EAT might protect the heart in low‐temperature conditions by providing heat to the myocardium. This is mainly based on the finding that UCP1, the main marker of brown adipose tissue, is relatively abundant in EAT.^34^, ^35^ However, this protective role remains controversial as the heart might generate enough heat from the breakdown of ATP. Second, EAT is thought to be an essential source of FFAs for cardiomyocytes. FFAs are the main energy substrate of cardiac energy metabolism under physical conditions, providing approximately 60−70% of energy production.^36^, ^37^, ^38^ Gas liquid chromatography analysis showed that EAT contains higher levels of saturated fatty acids than SAT. Furthermore, an in vitro study indicated that EAT has a higher rate of fatty acid incorporation and lipogenesis compared with other adipose depots.^39^, ^40^ These findings suggest that EAT can act as a local FFA storage. Third, EAT may also provide mechanical support to the heart and coronary arteries. In addition, EAT is an active endocrine organ that secretes numerous adipokines, including adiponectin, leptin, omentin‐1, and a range of inflammatory factors.^41^ Through the paracrine pathway, the secreted molecules can directly diffuse onto the underlying myocardium and coronary arteries.

In summary, the unique anatomy and physiology of the EAT determines its great potential in locally influencing adjacent tissues such as the myocardium and coronary arteries. EAT plays a unique role in the occurrence and development of several CVDs compared with the roles of VAT in general, which is a burgeoning research direction.^16^, ^29^, ^42^, ^43^

## 3 THE ASSOCIATION OF EAT WITH CVD AND METABOLIC DISEASE: CLUES FROM STUDIES BASED ON IMAGING TECHNIQUES

### 3.1 Medical imaging techniques used for measuring EAT

**FIGURE 2:**
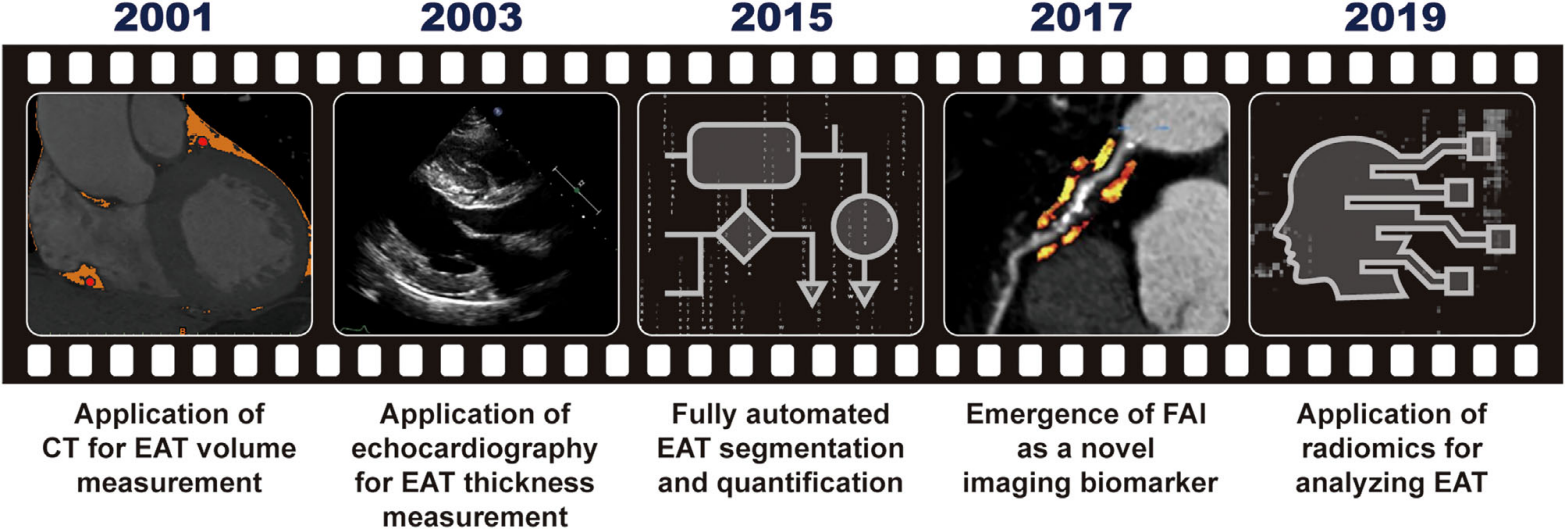
Milestones in the development of imaging techniques used in EAT‐related research. In 2001, Taguchi et al. first proposed a CT‐based measurement of EAT volume. In 2003, Iacobellis et al. first proposed using echocardiography to measure EAT thickness. Around 2015, algorithms for fully automated EAT segmentation and quantification emerged. In 2017, Antonopoulos et al. first proposed fat attenuation index of EAT as a novel imaging biomarker. In 2019, Oikonomou et al. first applied radiomics for analyzing EAT.

The difficulty of collecting EAT samples from patients and the absence of EAT in laboratory rats and mice led to a challenge in the field of EAT research.^30^ The progress of imaging techniques that provide new noninvasive methods for the measurement of EAT was a cornerstone in this area, which provided a powerful tool to study the correlations between EAT and CVDs (Figure 2). Therefore, studies based on imaging techniques hold an important position in EAT research and have made a significant contribution to our understanding of EAT.

Currently, three imaging techniques are widely used to assess EAT, including echocardiography, CT, and magnetic resonance imaging (MRI). In 2003, Iacobellis et al.^44^, ^45^ first proposed using transthoracic echocardiography to measure EAT thickness on the free wall of the right ventricle and pointed out that EAT thickness can be a new indicator of cardiovascular risk stratification. Soon, echocardiography became the most widely used method to measure EAT, owing to its accessibility and low cost. However, as pointed out by numerous researchers, this method has some drawbacks. First, echocardiography does not measure the entire volume of the EAT and only measures the EAT thickness at particular locations. Since EAT distribution is not uniform among patients, compared with EAT volume, EAT thickness is a less reliable measure of EAT amount. Accordingly, there is a stronger association between EAT volume and the occurrence of CVDs.^46^, ^47^ Second, the application of echocardiography is still restricted by its inability to differentiate between pericardial and epicardial fat.^48^, ^49^ Third, the reproducibility and precision of echocardiography is weaker than that of CT‐based methods.

In 2001, Taguchi et al.^50^ first proposed a CT‐based measurement of EAT volume. In this regard, the EAT area was manually traced on each slice of the CT scan, from the atrial appendage to the apex over the diaphragm, and the EAT volume was calculated as the sum of the EAT area. However, because this method was labor intensive and time consuming, it was not widely used. It should be noted that the CT‐based method of measuring EAT volume has been developed in line with the advancement of CT techniques and algorithms and has become more precise and practical.^51^ For instance, Park et al.^52^ introduced a threshold‐based three‐dimensional segmentation method for calculating EAT volume in 2010, which was found to be much more efficient than the conventional two‐dimensional short‐axis‐based approach. Since 2015, researchers have been exploring the application of computer algorithms for fully automated EAT segmentation and quantification.^53^, ^54^, ^55^, ^56^ After numerous upgrades and iterations, the related algorithms have matured substantially over the past decade and have been put into actual use in clinical studies exploring the role of EAT.^57^, ^58^, ^59^ The success of fully automated algorithms for EAT segmentation and quantification not only substantially reduced the workload required for EAT volume measurement (from more than 1 h to less than 1 min), but also improved accuracy in comparison with manual measurements. This remarkable progress technically paved the way for the clinical use of EAT volume as an indicator of cardiovascular risk.

In addition to its volume, average CT attenuation of the EAT, particularly the perivascular portion, which is termed the perivascular fat attenuation index (FAI), has emerged as a novel imaging biomarker and has gained great attention in the last few years.^60^, ^61^, ^62^, ^63^, ^64^, ^65^ FAI has been demonstrated as a new sensitive imaging marker in describing coronary inflammation.^60^, ^66^ In recent years, CT‐based method has become the gold standard of EAT volume measurement and been used more and more often owing to the continuous improvement of its accuracy and convenience. Similar to CT, cardiac MRI can provide volumetric measurements of EAT. However, MRI measurements of EAT are limited owing to higher cost, less availability, and long postprocessing time.^67^

The utilization of radiomics in the analysis of EAT represents another significant advancement in the imaging modalities employed in EAT‐related research. Radiomics was first described in the literature around 2012 and was mainly used for CT‐image analysis of tumors.^68^, ^69^, ^70^ In 2019, radiomics was first used for analyzing EAT by Oikonomou et al.,^71^ which substantially increased the amount of quantitative information accessible from CT images. In traditional ways, the evaluation indicators of EAT that could be obtained from CT images were very limited, with only quantitative indexes (volume or thickness) and average CT attenuation. It should be noted that a wealth of information contained in CT images other than these indexes was neglected. In contrast, radiomics is capable of extracting thousands of features, including geometric structure, texture, intensity distribution, and so on from medical images, subsequently converting them into minable data, which can then be analyzed by computational techniques, including machine learning, deep learning, and so on.^72^, ^73^ Thus, radiomics approaches can identify novel imaging patterns that are indiscernible to the human eye, and thereby maximize available data of CT images. Furthermore, the image patterns of EAT identified by radiomics have a great potential to become novel indicators to predict the incidence or prognosis of CVDs. However, routine application of radiomics analysis in clinical settings remains challenging owing to the lack of reproducibility as the analysis process is susceptible to a variety of technical factors.^74^, ^75^, ^76^ Nevertheless, radiomics is an exciting technological trend of EAT‐related research based on imaging techniques.

### 3.2 Association between increased EAT amount and CVDs in patients with metabolic disorders

#### 3.2.1 Increased EAT in obese or diabetic patients indicates a higher CVD risk

Numerous studies have revealed a strong linear association between the EAT amount and obesity.^77^, ^78^, ^79^ Similarly, EAT thickness and volume were significantly higher in patients with type 2 diabetes mellitus (T2DM).^77^, ^80^, ^81^, ^82^ Additionally, an increased EAT is also an indicator of new‐onset diabetes mellitus. In a retrospective study, Kang et al.^83^ found that patients with an EAT thickness ≥5 mm at baseline had a significantly higher risk of developing diabetes. EAT is also associated with other components of metabolic syndrome (MetS). Several studies have shown that a graded relationship exists between the number of MetS components and EAT thickness.^80^, ^84^ EAT is considered a useful indicator of the risk of developing MetS as well.

In the diabetic population, an expanded EAT has been suggested to play a role in accelerating CVDs and increasing mortality. In this regard, through logistic regression analysis, Wang et al.^80^ found that the EAT volume was significantly associated with the Gensini score, coronary lesions, and coronary calcium scores in patients with T2DM. Similarly, according to a prospective study that included 200 patients with T2DM but without a known coronary artery disease (CAD), a high EAT volume was associated with an increased risk of CAD incident after a 6‐year follow‐up.^85^ Other researchers have reported similar findings.^58^, ^86^, ^87^ In addition to EAT volume, the latest research shows that radiomics parameters of EAT, especially pericoronary adipose tissue (PCAT), are promising tools in cardiovascular risk evaluation in patients with MetS. According to Shang et al.,^88^ radiomics‐based assessment of PCAT is clearly superior to plaque scores in predicting future acute coronary syndrome within three years. Furthermore, evidence has shown that models encapsulating radiomics features of PCAT and clinical factors are valuable in the early diagnosis of CAD in patients with T2DM.^89^

Moreover, the amount of EAT is associated with myocardial function in T2DM patients. Accordingly, it has been reported that diastolic function is worse in diabetic patients with higher EAT thickness.^90^, ^91^ However, this conclusion remains controversial and is still under debate, as some researchers have not observed an association between EAT volume and myocardial perfusion or myocardial microvascular function in patients with either type 1 or type 2 diabetes.^92^, ^93^, ^94^

Overall, mounting evidence has proven that the amount of EAT increases in obese and diabetic patients. In the diabetic population, a higher EAT volume is associated with an increased risk of CVDs and reduced cardiac diastolic function. However, further in‐depth investigations are still needed to explore whether T2DM aggravates the pathogenic potential of EAT and whether EAT plays a role in accelerating the progression of CAD in diabetic patients.

#### 3.2.2 Association between EAT amount and CVD biomarkers in patients with metabolic disorders

In addition to studies evaluating the role of EAT amount in predicting the incidence and prognosis of CVDs, others explored the association between EAT amount and expression level of CVD biomarkers.

Demir et al.^95^ found that oxidative stress parameters, especially serum levels of total oxidant status, were higher in patients with MetS compared with the control group. EAT thickness measured by transthoracic echocardiography is also higher in the MetS group and was significantly positively correlated with the level of total oxidant status. Similarly, the same group reported a significant positive correlation between serum IL‐17A levels and EAT thickness in patients with MetS.^96^

Several studies have shown that increased EAT thickness is strongly associated with elevated BNP/NT‐proBNP levels in patients with MetS.^97^, ^98^ Thus, evidence suggests that EAT thickness and BNP/NT‐proBNP levels can corroborate each other and be used for risk stratification of patients with MetS. However, we must acknowledge that such kind of studies cannot draw any conclusions about causality between EAT amount and other biomarkers.

#### 3.2.3 EAT as a possible explanation for the increased prevalence of atrial fibrillation in obese patients

Mounting evidence has shown that obesity is a major risk factor for atrial fibrillation (AF), and EAT with increased volume in obese patients might partly account for this higher prevalence.^99^, ^100^ Studies based on imaging techniques have proven that the EAT volume is highly associated with AF, even after adjustment for risk factors such as left atrial enlargement and body mass index (BMI).^101^, ^102^, ^103^ Additionally, Klein et al.^104^ found that low left atrial EAT attenuation measured by CT is associated with the presence of a low‐voltage zone within the adjacent myocardium, which is thought to be associated with fibrosis. EAT volume is also associated with AF ablation outcomes. Based on the study by Beyer et al.,^105^ EAT volume is significantly higher in patients with AF recurrence compared with those without AF recurrence (144.5 vs. 128.5 mm^3^; *p* < 0.0001). Moreover, lower EAT attenuation levels indicating a higher lipid component is also associated with AF recurrence.^105^, ^106^ In addition, recent studies have demonstrated a significant difference in the radiomics phenotype of EAT between patients with lone AF and the control group, and even between different AF subtypes.^107^, ^108^ Furthermore, some specific radiomics features, such as Gray‐Level Size Zone Matrix, can act as an independent predictor of AF recurrence, which might be of practical clinical value in AF management.^108^, ^109^

Considering the aforementioned, studies based on imaging techniques have clearly suggested a close relationship between EAT and AF. These encouraging results have stimulated further interest in the investigation of the mechanism linking EAT to AF, which will be further discussed in the following section.

In summary, studies based on imaging techniques have made important contributions to deepening our understanding of the key role of EAT in CVDs (Table 1). However, intrinsic defects exist in all studies based on imaging techniques. In this regard, the measurable parameters of EAT are still very limited. It should be noted that a single parameter of EAT amount alone, either EAT thickness or EAT volume, cannot represent the properties of EAT. Additionally, an increased amount of EAT does not necessarily mean a higher degree of inflammation.^15^ The EAT secretory phenotype may be quite different even in patients with similar EAT amounts. The other shortfall of studies based on imaging techniques is that they can only demonstrate a correlation between EAT and CVDs, and it is difficult to establish causal conclusions based on them. To further understand the mechanisms by which EAT affects adjacent structures, such as the coronary artery and myocardium, direct in‐depth analysis of clinical EAT specimens is indispensable.

**TABLE 1:** Studies based on imaging techniques exploring the association between EAT, metabolic disorders, and CVDs.

Study	Year	Study design	Study population and sample size	Method	Major findings
Wang et al.^80^	2009	Cross sectional	T2DM patients (*n* = 49); nondiabetic patients (*n* = 78)	CT: EAT volume	EAT volume was higher in T2DM patients than nondiabetic patients. EAT volume was associated with unfavorable components of MetS and coronary atherosclerosis.
Tonbul et al.^81^	2011	Cross sectional	End‐stage renal disease (ESRD) patients: T2DM (*n* = 17); nondiabetic (*n* = 43)	CT: EAT volume	EAT volume was higher in ESRD patients with T2DM than those without T2DM.
Cetin et al.^197^	2013	Cross sectional	T2DM patients (*n* = 139); nondiabetic patients (*n* = 40)	Echocardiography: EAT thickness	EAT thickness was higher in T2DM patients than nondiabetic patients. EAT thickness was significantly associated with waist circumference and carotid intima‐media thickness.
Yorgun et al.^84^	2013	Cross sectional	MetS patients: (*n* = 40); non‐MetS patients: (*n* = 43)	CT: EAT thickness	EAT thickness was higher in MetS patients than non‐MetS patients.
Mun˜oz et al.^198^	2014	Cross sectional	Postmenopausal women: MetS (*n* = 14); non‐MetS (*n* = 20)	Echocardiography: EAT thickness	EAT thickness was higher in postmenopausal women with MetS than those without MetS. EAT thickness was positively correlated with visceral adipose tissue, BMI, waist circumference in postmenopausal women.
Akbas et al.^199^	2014	Cross sectional	T2DM patients (*n* = 156); nondiabetic patients (*n* = 50)	Echocardiography: EAT thickness	EAT thickness was higher in T2DM patients than nondiabetic patients. EAT thickness was positively correlated with waist circumference and platelet‐to‐lymphocyte ratio in T2DM patients.
Groves et al.^82^	2014	Cross sectional	Asymptomatic T2DM patients without known CAD (*n* = 92); asymptomatic nondiabetic patients (*n* = 270)	CT: EAT volume	EAT volume was higher in T2DM patients than nondiabetic patients. EAT was an independent predictor of the incidence and severity of CAD.
Song et al.^77^	2015	Cross sectional	94 Patients (40 with T2DM, 50 with obesity)	CT: EAT thickness and epicardial fat area (EFA) measured at the level of left main coronary artery	EAT thickness and EFA was higher in T2DM patients than nondiabetic patients. EAT thickness and EFA was higher in obese patients than nonobese patients.
Kaya et al.^200^	2015	Cross sectional	Geriatric patients: MetS (*n* = 30); non‐MetS (n = 30)	Echocardiography: EAT thickness	EAT thickness was higher in geriatric MetS patients than non‐Met patients.
Evin et al.^91^	2016	Cross sectional	Patients with obesity and T2DM (*n* = 20); healthy controls without obesity or T2DM (*n* = 19)	MRI: EAT volume	EAT volume was higher in patients with obesity and T2DM than healthy controls.
Christensen et al.^85^	2017	Prospective	Patients with T2DM and elevated urinary albumin excretion rate (UAER) (*n* = 200)	Echocardiography: EAT thickness	Increased EAT thickness was associated with increased risk of incident CVDs or all‐cause mortality in patients with T2DM and elevated UAER.
Chen et al.^201^	2017	Cross sectional	T2DM patients (*n* = 167); nondiabetic patients (*n* = 82)	Echocardiography: EAT thickness	EAT thickness was higher in T2DM patients than nondiabetic patients. EAT thickness was independently associated with serum 25‐hydroxyvitamin D and HOMA‐IR.
Seker et al.^202^	2017	Prospective	Patients with non‐ST elevation myocardial infarction: T2DM (*n* = 186); nondiabetic: (*n* = 268)	Echocardiography: EAT thickness	EAT thickness was higher in T2DM patients than nondiabetic patients. EAT thickness was independently associated with extent and complexity of CAD (evaluated by SYNTAX score).
Wang et al.^203^	2017	Cross sectional	T2DM patients (*n* = 68); nondiabetic patients (*n* = 30)	Echocardiography: EAT thickness	EAT thickness was higher in T2DM patients than nondiabetic patients. EAT thickness was significantly associated with intimal medial thickness of carotid artery.
Kang et al.^83^	2018	Retrospective	Patients with CAD treated with high‐intensity statins (*n* = 321)	Echocardiography: EAT thickness	Epicardial adipose tissue thickness is a consistent independent predictor of new‑onset T2DM in patients with CAD treated with high‐intensity statins.
Christensen et al.^86^	2019	Prospective	T2DM patients (*n* = 1030)	Echocardiography: EAT thickness	High levels of EAT were associated with increased incident CVD and mortality in patients with T2DM.
Lin et al.^58^	2021	Prospective	MetS patients (*n* = 280); non‐MetS patients (*n* = 1788)	CT: EAT volume and EAT attenuation	EAT thickness was higher and EAT attenuation was lower in MetS patients than non‐MetS patients.
Ichikawa et al.^204^	2022	Prospective	T2DM patients (*n* = 333)	CT: LAD‐PCAT attenuation	LAD‐PCAT attenuation was significantly higher in patients with cardiovascular events than in those without.

Abbreviations: CAD, coronary artery disease; EAT, epicardial adipose tissue; ESRD, end‐stage renal disease; LAD, left anterior descending coronary artery; MetS, metabolic syndrome; PCAT, paracardial adipose tissue.; T2DM, type 2 diabetes mellitus.

## 4 EAT REMODELING IN CARDIOMETABOLIC CONDITIONS AND CVDS: KNOWLEDGE FROM IN‐DEPTH PROFILING OF EAT

Studies based on imaging techniques have provided valuable data indicating a close association between EAT amount, metabolic diseases, and CVDs. However, it is far from enough to depict a patient's EAT with only measurements based on imaging techniques. Accordingly, an in‐depth investigation of the EAT properties is necessary. In patients with similar EAT volumes, the immune profile, inflammation state, and secretome of the EAT can vary substantially. In recent years, the advancement of omics technologies has provided powerful tools to profile EAT in depth, allowing us to gain deeper insights into the association between EAT properties and CVDs. Numerous efforts have been devoted to investigating the profile of EAT in different patient populations and have profoundly expanded our knowledge of EAT remodeling in obese and diabetic patients beyond volume changes. Key studies exploring the profile of EAT and its secretome are summarized in Table 2. Furthermore, this review provides a summary of the key cytokines and adipokines secreted by EAT, along with their respective functions, which can be found in Table 3.

**TABLE 2:** Studies exploring the phenotype of EAT.

Study	Year published	Experimental vs. control groups	Sample collection	Detecting method	Involved adipocytokines or cytokines	Major findings
Mazurek et al.^41^	2003	EAT vs. SAT (*n* = 42, paired samples)	EAT and SAT samples, collected form patients undergoing CABG surgery	RT‐PCR, ELISA	IL‐1β, IL‐6, MCP‐1, and TNF‐α	The levels of IL‐1β, IL‐6, MCP‐1, and TNF‐α in EAT were significantly higher than SAT.
Iacobellis et al.^205^	2005	CAD (*n* = 16) vs. non‐CAD (*n* = 6)	EAT samples collected form CAD‐patients (undergoing CABG surgery) and non‐CAD patients (undergoing valve replacement or atrium septal defect repair)	Western blot	Adiponectin	The level of adiponectin EAT was significantly lower in CAD‐patients than non‐CAD patients.
Baker et al.^206^	2006	EAT (*n* = 46) vs. omental fat (*n* = 14) vs. abdominal SAT (*n* = 30) vs. gluteal SAT (*n* = 13)	EAT, omental fat, abdominal SAT, and gluteal SAT samples, collected from patients undergoing CABG surgery	RT‐PCR	Resistin, adiponectin	The level of resistin in EAT was threefold higher than that in gluteal adipose tissue. The level of adiponectin EAT is significantly lower than that in omental fat, abdominal SAT, and gluteal SAT.
Kremen et al.^207^	2006	Preoperative vs. postoperative (*n* = 15, paired samples)	EAT and SAT samples, collected at the beginning (considered as preoperative) and before the end (considered as postoperative) of elective cardiac surgery (10 CABG surgeries and 5 valvular plastique)	RT‐PCR	Leptin, adiponectin, CD14, CD68, Resistin, MCP‐1, IL‐6	The levels of IL‐6, resistin, and MCP‐1 in both EAT and SAT significantly increased at the end of the surgery. The levels of Leptin, adiponectin, CD14, and CD68 did not change significantly.
Cheng et al.^208^	2008	CAD (*n* = 46) vs. non‐CAD (*n* = 12)	EAT and abdominal adipose tissue samples, collected from CAD‐patients (undergoing CABG surgery) and non‐CAD patients (undergoing valve replacement or atrium septal defect repair)	ELISA	TNF‐a, IL‐6, leptin, visfatin, adiponectin	The levels of TNF‐a, IL‐6, leptin, and visfatin in conditioned media of EAT form CAD patients were significantly higher than non‐CAD patients. The level of adiponectin in conditioned media of EAT form CAD patients was significantly lower than non‐CAD patients.
Eiras et al.^209^	2008	CAD (*n* = 58) vs. non‐CAD (*n* = 34)	EAT and SAT samples, collected form CAD‐patients (undergoing CABG surgery) and non‐CAD patients (undergoing valve surgery)	RT‐PCR	Adiponectin, IL‐6	EAT of CAD patients expressed higher level of IL‐6 and lower level of adiponectin compared with non‐CAD patients. Increased extension of CAD is significantly associated with higher level of IL‐6 and lower level of adiponectin in EAT.
Eiras et al.^210^	2010	EAT vs. SAT (*n* = 55, paired samples)	EAT and SAT samples, collected from patients undergoing elective cardiac surgery (CABG or valve replacement)	Nitroblue tetrazolium chloride assays (for measuring ROS)	Production of reactive oxygen species (ROS)	The level of oxidative stress is greater in EAT than SAT in patients with CVDs.
McAninch et al.^111^	2015	EAT vs. SAT (*n* = 23, paired samples)	EAT and SAT samples, collected form CAD‐patients (undergoing CABG surgery) and non‐CAD patients (undergoing valve surgery)	Transcriptome		The EAT transcriptome is distinct form SAT. Relative to SAT, EAT is a highly inflammatory tissue.
Du et al.^211^	2016	CAD (*n* = 28) vs. non‐CAD (*n* = 12)	EAT and SAT samples, collected form CAD‐patients (undergoing CABG surgery) and non‐CAD patients (undergoing valve surgery)	RT‐PCR	Omentin‐1, adiponectin	The levels of omentin‐1 and adiponectin in EAT of CAD patients were significantly lower than non‐CAD patients. In CAD patients, omentin‐1 expression was lower in EAT surrounding coronary segments with stenosis than those without stenosis.
Gruzdeva et al.^212^	2017	EAT vs. SAT (*n* = 24 paired samples)	EAT and SAT samples, collected form patients undergoing CABG surgery	ELISA	Leptin, TNF‐α, IL‐1, adiponectin, IL‐10, FGF‐β	The levels of leptin, soluble leptin receptor, TNF‐α, and IL‐1 were higher in cultured adipocytes form EAT than SAT. The levels of anti‐inflammatory cytokines, including adiponectin, IL‐10 and FGF‐β were lower in cultured adipocytes form EAT than SAT.
Gruzdeva et al.^213^	2019	EAT vs. SAT (*n* = 84, paired samples) Subgroup: Obesity (*n* = 54) vs. nonobesity (*n* = 30)	EAT and SAT samples, collected form patients undergoing CABG surgery	RT‐PCR	Adiponectin, IL‐10, leptin, IL6, TNF‐α	The levels of adiponectin and IL‐10 were lower in cultured adipocytes form EAT than SAT. The levels of leptin, IL6, and TNF‐α were higher in cultured adipocytes form EAT than SAT. The level of adiponectin was lower in cultured EAT and SAT adipocytes form obese patients than nonobese patients.
Sardu et al.^214^	2019	Prediabetic patients (*n* = 180) vs. normoglycemic (NG) patients (*n* = 180) (matched patients)	EAT and SAT samples, collected form patients undergoing CABG surgery	ELISA	Proinflammatory tone, defined as TNF‐α, reduced SIRT6 levels, and leptin to adiponectin ratio	The level of inflammatory tone was higher in prediabetic patients than NG‐patients and was highly associated with the MACE during the 12‐months follow‐up.
Zhang et al.^215^	2019	CAD (*n* = 38) vs. non‐CAD (*n* = 40)	EAT and SAT samples, collected form CAD‐patients (undergoing CABG surgery) and non‐CAD patients (undergoing valve surgery or atrium septal defect repair)	RT‐PCR, immunohistochemistry	Leptin	The level of leptin in EAT of CAD‐patients was significantly higher than non‐CAD patients. In subgroup analysis, the level of leptin in EAT of CAD‐patients with local coronary stenosis near the right coronary artery ostium was significantly higher than those without.

Abbreviations: CABG, coronary artery bypass grafting; CAD, coronary artery disease.; EAT, epicardial adipose tissue; MCP‐1, monocyte chemoattractant protein‐1; RT‐PCR: real‐time polymerase chain reaction, ELISA: enzyme‐linked immunosorbent assay; SAT, subcutaneous adipose tissue; TNF‐α, tumor necrosis factor‐α.

**TABLE 3:** EAT derived adipocytokines and cytokines.

Role	Factor	Major functions	References
Protective/anti‐inflammatory	Adiponectin	Anti‐inflammatory, antifibrotic, antiapoptotic, and antioxidative effects Cardiovascular protective effects Enhancing insulin sensitivity	^205^, ^206^, ^207^, ^209^, ^211^, ^214^, ^216^, ^217^, ^218^
	Omentin‐1	Anti‐inflammatory, antioxidative, antiapoptotic effects. Antiatherosclerosis and cardiovascular protective effects Suppressing insulin resistance	^211^, ^219^, ^220^
	IL‐10	Anti‐inflammatory effects	^213^, ^221^, ^222^
Pathologic/proinflammatory	Leptin	Regulating neuroendocrine function and energy homeostasis	^207^, ^208^, ^214^, ^215^, ^216^, ^223^, ^224^
	TNF‐α	Proinflammatory effects Inhibiting adiponectin secretion	^41^, ^213^, ^225^, ^226^
	IL‐1		^41^, ^227^
	IL‐6		^209^, ^228^, ^229^
	IL‐8		^230^
	Resistin	Proinflammatory effects Involved in insulin resistance, tumorigenesis	^206^, ^207^, ^231^, ^232^, ^233^

The inflammatory phenotype of EAT has been the focus of related studies. Our earliest recognition of its inflammatory phenotype came from a classic study by Mazurek et al.^41^ Through analysis of EAT samples obtained from patients who underwent elective coronary artery bypass graft (CABG) surgery, they found that EAT from patients (*n* = 42) with CAD expressed significantly higher levels of proinflammatory cytokines (tumor necrosis factor‐α [TNF‐α], interleukin‐6 [IL‐6], and IL‐1β) compared with those produced by SAT from the same patient, which was confirmed by reverse transcription PCR and ELISA results.^41^ Additionally, they reported that the degree of inflammatory cell infiltration was also higher in EAT than in SAT.

Subsequently, numerous studies have further investigated the inflammatory profile of EAT, making efforts to find its association with CVDs, among which CAD has gained much attention. In many studies, EAT samples obtained from patients who underwent non‐CABG surgeries were selected as a control group for the CAD group to explore whether the EAT phenotype is associated with CAD. Sacks et al.^110^ showed that the expression levels of proinflammatory and redox genes in EAT from patients with severe CAD were significantly higher than in those from patients without CAD. Another study by McAninch et al.^111^ evaluated the transcriptome of EAT and SAT samples from patients who underwent CABG (*n* = 6) or cardiac valve replacement surgery (*n* = 5) and found that the EAT transcriptome is markedly different from SAT, exhibiting a high inflammatory phenotype. However, the difference in the inflammatory phenotype between the CAD and non‐CAD groups was not significant. In this regard, as shown by other studies, although it is widely recognized that the inflammatory phenotypes of EAT and SAT are remarkably different, whether CAD status is independently associated with significant alterations in the inflammatory profile of EAT remains controversial.^20^, ^112^, ^113^ The lack of an ideal control group is a major issue in such studies and might be responsible for the controversy.

Owing to the limitations of realistic factors, EAT samples used for research can only be obtained from elective cardiac surgeries. Consequently, it is difficult to identify an appropriate control group with the same baseline characteristics for patients who undergo CABG surgery. For instance, patients undergoing cardiac valve replacement surgery are the most commonly used control group. However, the patient characteristics in the CAD and valvulopathy groups show obvious differences. These differences can be explained by the fact that individuals with cardiovascular risks, such as obesity and diabetes, are much more likely to suffer from severe CAD. Accordingly, patients undergoing CABG typically have a higher age and prevalence of obesity, dyslipidemia, hypertension, and diabetes than patients undergoing cardiac valve replacement surgery.^41^, ^111^, ^112^ Therefore, to eliminate the interference of different baseline data, patients enrolled in the control group should be carefully selected to match those in the CAD group. Otherwise, no definitive conclusion can be drawn that CAD status is independently associated with the inflammatory profile of EAT.

Some researchers have taken another track to investigate the association between cardiometabolic conditions, such as obesity and diabetes, and the EAT profile. They pointed out that the underlying cardiometabolic conditions of the patients, rather than the cardiac disease phenotype, are more central in determining the inflammatory profile of EAT.^20^

In the study by Fitzgibbons et al.,^113^ the baseline data were well matched between the CAD (*n* = 13) and non‐CAD (*n* = 13) groups. Accordingly, by analyzing the transcriptome of EAT samples, they found no difference in the expression levels of proinflammatory cytokines such as chemokine (C‐C motif) ligand 2 (CCL2), C‐C chemokine receptor type 2 (CCR2), TNF‐α, IL‐6, IL‐8, and plasminogen activator inhibitor‐1 between the two groups.^113^ In contrast, even in the non‐CAD group, EAT compared with SAT had a higher level of inflammatory gene expression. Thus, they concluded that the CAD disease status is not highly associated with the EAT profile. Consistent with this view, Vyas et al. ^20^ demonstrated that the inflammatory profile of the EAT is independent of CAD severity, but underlying cardiometabolic conditions, especially obesity and diabetes, are major risk factors for EAT inflammation. Through flow cytometry analysis, they found no difference in key immune cell numbers or inflammatory mediator levels in the EAT between the CABG and valvular replacement groups. Further, Fitzgibbons et al.^113^ redivided patients enrolled into three groups according to their cardiometabolic conditions including lean nondiabetic (BMI < 25) (*n* = 30), obese nondiabetic (BMI ≥ 25), and obese (BMI ≥ 25) type 2 diabetic patients. Although the immune cell infiltration in EAT was similar across groups, obese and T2DM patients showed dramatically higher levels of proinflammatory cytokines such as IL‐1, IL‐6, TNF‐α, and interferon‐gamma than lean nondiabetic patients.^20^ Further, subsequent bulk RNA‐seq confirmed this finding. In addition to the remarkably upregulated inflammatory genes, the metabolic phenotype of EAT was profoundly remodeled in obese and diabetic patients. Moreover, the expression patterns of the genes involved in glucose and lipid metabolism were downregulated in the same group.

Another study classified patients with ischemic heart disease (IHD) who had undergone CABG surgery into two groups according to their T2DM status: IHD‐T2DM group (*n* = 23) and IHD‐NoT2DM group (*n* = 22).^114^ The control group included patients who underwent valve replacement surgery and did not have IHD or T2DM. The authors measured the mRNA expression of scavenger receptors (SRs), such as LOX‐1 and CL‐P1, which are thought to play a pivotal role in the formation of atherosclerotic plaques. A significant difference in SR expression levels was observed between the IHD‐T2DM and IHD‐NoT2DM groups (*p* < 0.01), but not between the IHD‐NoT2DM and control groups. Macrophage infiltration was also higher in the IHD‐T2DM group.^114^ In line with previous studies, this study further demonstrated the essential role played by cardiometabolic conditions in the profile of EAT, rather than CAD status. In this regard, other studies have reported similar results.^115^, ^116^, ^117^

Considering the above, there is a growing body of evidence suggesting that cardiometabolic conditions, such as obesity and diabetes, are the major determinants of the inflammatory profile of EAT, while it is still controversial whether CAD is independently associated with the EAT profile. This prompts us to consider that EAT with proinflammatory phenotype may serve as a local mediator of the deleterious effects of cardiometabolic conditions. These valuable findings have also motivated the great interest of researchers to further validate the association between the EAT phenotype, metabolic disorders, and CVDs, together with the underlying mechanisms.

## 5 REMODELED EAT MIGHT BE A CAUSE OF CVDS: PRELIMINARY EVIDENCE FROM IN VITRO COCULTURE STUDIES

In comparison with the studies based on imaging techniques, profiling studies provide much deeper insights into the EAT phenotype and its association with CVDs and cardiometabolic conditions. Although imaging studies are based on EAT volume whereas profiling studies are based on omics data, these two types of studies are essentially the same, considering both are correlational studies. In this regard, neither of them can establish a causal relationship between the properties of EAT and CVDs. However, our exploration of EAT cannot stop here. Further studies are required to validate whether a causal association exists between EAT and CVDs.

Given the limitations of realistic factors, it is still impractical to perform RCTs on patients to prove that dysfunctional EAT accelerates CAD progression. In addition, the absence of EAT in laboratory rats and mice made them difficult to use for EAT‐related studies, which may explain why only a few basic studies and almost no animal studies on EAT are available. In this context, some researchers have proposed an alternative to explore the causal link between EAT and CVDs by designing coculture experiments. In this regard, the paracrine effect of EAT can be studied by incubating cell models of CVDs with secretory products generated from explants of human EAT samples. Currently, the conditioned media (CM) of EAT tissue samples is the most frequently used secretory product.^118^, ^119^, ^120^, ^121^ Considering that the molecules secreted by adipose tissue can be encapsulated in extracellular vesicles (EVs), EVs isolated and purified from the CM of EAT have also been studied as a more specific paracrine mechanism.^21^, ^122^ Further, EAT blocks can also be directly cocultured with cardiomyocytes to study their paracrine effect.^123^ By combining the in‐depth profiling of EAT and its secretory products, researchers can predict the possible active ingredients in the secretome and finally verify its role in in vitro or in vivo models. Although the conclusions are reached with cell models, a causal relationship can be established between EAT and CVDs, which can compensate for the defects of correlational studies to some extent.

However, we need to be cognizant of the fact that compared with studies based on imaging techniques, studies exploring the mechanism of action of EAT remain scarce. Besides, as the components of EAT secretome are complex, it is hard to conclude that certain molecular or pathway is the primary mechanism of harmful/protective effect of EAT. Moreover, to what extent do the secretory products of EAT generated in vitro can be representative of those in vivo remains to be determined. Overall, the investigations about the mechanism of action of EAT are still in their infancy, additional research and better experimental design is warranted.

### 5.1 Secretory products of EAT from diabetic patients impair myocardial function

Greulich et al.^118^ carried out a series of studies using CM generated from explants of EAT tissue samples, and made great contributions to our understanding of the potential causal link between dysfunctional EAT of diabetic patients and impaired cardiac function.^120^ They obtained EAT and SAT biopsies from patients who underwent CABG or valve replacement surgery and divided the patients into two groups according to their T2DM status. Accordingly, adipose tissue samples were cultured in adipocyte media without serum for 24 h; then, the CM of fat tissue was collected for the experiment. Further, primary rat cardiomyocytes were incubated in the CM for 30 min. Compared with CM‐EAT from nondiabetic patients (CM‐EAT‐ND) and CM‐SAT, CM‐EAT from patients with T2DM (CM‐EAT‐DM2) significantly impaired the contractile function of cardiomyocytes. To identify the factors responsible for the deleterious effect in CM‐EAT‐DM2, the authors profiled the CM using an antibody array and found that the secretory profile of EAT‐DM2 was considerably different from that of EAT‐ND. Accordingly, they revealed that activin A was one of the differentially expressed molecules, and a neutralizing antibody against activin A alleviated the deleterious effect of CM‐EAT‐DM2. Further, direct exposure of cardiomyocytes to activin A mimicked the detrimental effects of CM‐EAT‐DM2, indicating that activin A is a major active ingredient in the EAT secretory product. This study further demonstrated the dramatic remodeling of the EAT secretome in diabetic patients and proposed that activin A may be partly responsible for the detrimental effects of EAT.^118^ Further, using a similar approach, the group demonstrated that the RAS/miR‐208a pathway is also involved in the impairment of cardiomyocyte contractile function induced by CM‐EAT‐DM2.^120^

Another study by Akawi et al.^23^ showed that obesity is associated with dysregulation of the metabolome of the PAT. The authors used CM collected from cultured adipose biopsies to analyze the metabolites secreted by PAT and SAT and found a significant difference in sphingolipid (SPL) secretion. Moreover, they revealed that PAT from obese patients secreted a higher amount of SPL than propensity‐matched nonobese patients. To investigate the paracrine effect of PAT on vascular endothelial cells, C16:0‐ceramide (Cer16:0), the most abundant SPL generated by PAT, was used for in vitro experiments. They found that Cer16:0 can be effectively taken up by immortalized human aortic endothelial cells (HAECs) and cause dramatic O_2_^.−^ production in cells. Furthermore, clinical data have shown that increased PAT SPL secretion is closely correlated with increased O_2_^.−^ levels and impaired endothelial function in human vessels. In this regard, a 5‐year follow‐up found that patients with high circulating Cer16:0 levels have a twofold higher risk of fatal cardiac events.^23^ This study proposed a possible causal relationship between PAT, obesity, and endothelial function and suggested that the remodeled secretome of PAT in obese patients may exert adverse effects on adjacent vascular endothelial cells in a paracrine manner.

### 5.2 Exploring the causal link between EAT and AF

Evidence provided by studies based on imaging techniques strongly indicates that EAT may be involved in the occurrence and development of AF, and remodeled EAT may explain the high incidence rate of AF in obese patients. Whether a causal link exists between EAT and AF remains to be determined. Shaihov‐Teper et al.^21^ isolated and purified EVs from the culture media of EAT explants. Cytokine analysis found that EAT‐EVs from patients with AF contained higher amounts of proinflammatory and profibrotic cytokines than those from patients without AF. Moreover, proteomic analysis revealed a distinct proteome profile of EAT‐EVs from patients with AF. Several proteins related to atrial fibrosis, angiogenesis, apoptosis, and myopathy were exclusively expressed in EAT‐EVs isolated from patients with AF, but not in non‐AF patients. The authors further investigated the causal association between EAT‐EVs and AF using cell and animal models. Compared with those from non‐AF patients, EAT‐EVs from patients with AF promoted human right atrial mesenchymal stromal cell migration and gap closure in the scratch migration assay, exhibiting profibrotic effects. Furthermore, injection of EAT‐EVs from AF patients into the left ventricle of rats caused extensive myocardial fibrosis, whereas EAT‐EVs from patients with AF did not. Further, this in vivo experiment confirmed the profibrotic effects of EAT‐EVs in patients with AF. In addition, EAT‐EVs isolated from patients with AF had greater angiogenic capacity, as demonstrated by an angiogenic tube formation assay, which is related to the pathogenesis of AF. Although it is still in the stage of in vitro or animal experiments, this study provided direct evidence for the causal relationship between dysfunctional EAT and AF.

Other studies have also reached similar conclusions.^123^, ^124^ Based on CT measurements of EAT volume and cardiac electrophysiological mapping in patients, Nalliah et al.^123^ found that the right atrial EAT volume is highly associated with atrial electrophysiological dysfunction and cardiac fibrosis, which are related to the mechanisms underlying AF. Further, they performed in vitro coculture experiments to investigate the paracrine effects of EAT on cardiomyocyte electrophysiology. They cocultured ovine EAT with human‐induced pluripotent stem cell‐derived cardiomyocytes and observed changes in cardiomyocyte electrophysiology with a slower spontaneous beating rate and prolonged potential duration. Similarly, incubation of mouse HL‐1 atrial cardiomyocyte monolayers with CM from pericardial adipose tissue of mice fed a high‐fat diet led to a longer activation time and slower conduction velocity.^123^

Considering the above, although in vitro coculture studies are still in the stage of cell experiments and might be inadequate to entirely simulate (or be fully representative of) the paracrine effect of EAT in the human body, they have made an essential contribution to our understanding of the role played by EAT in CVDs. These studies have provided preliminary evidence that the paracrine effect of remodeled EAT on adjacent cardiomyocytes can promote the occurrence and progression of CVDs.

## 6 EXPLORATION OF TARGETING EAT AS A THERAPEUTIC STRATEGY

Owing to the rapid advancement in research, there is a growing appreciation that EAT may play an important role in CVDs.^125^, ^126^, ^127^, ^128^ This conclusion naturally leads to the great interest of researchers in targeting EAT as a therapeutic strategy to reduce cardiovascular risks.

**FIGURE 3:**
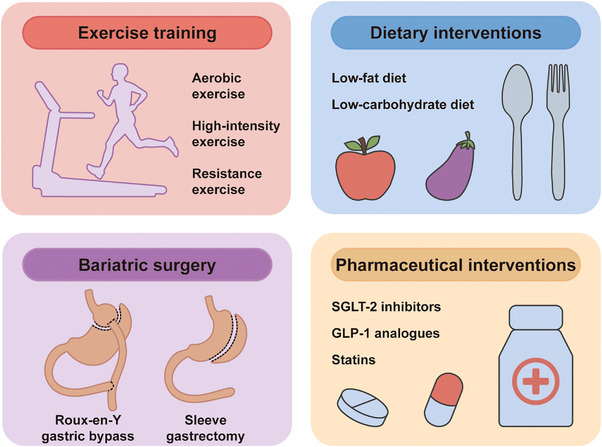
Interventions targeting epicardial adipose tissue (EAT). Four types of interventions have been proposed to reduce EAT volume, including exercise interventions, dietary interventions, bariatric surgery, and pharmaceutical interventions. Several types of exercise training protocols and dietary interventions have proven effective in reducing EAT amount. Besides, bariatric surgeries, including sleeve gastrectomy and Roux‐en‐Y gastric bypass, can lead to pronounced effects in decreasing EAT, as well as other fat deposits. Finally, drugs including sodium‐glucose cotransporter‐2 (SGLT‑2) inhibitors, glucagon‐like peptide‐1 (GLP‐1) analogues and statins have been studied to reduce EAT amount.

Nevertheless, related studies are still largely confined to exploring ways to reduce the amount of EAT, which appears to be the most straightforward strategy. In the past decade, four types of interventions have been proposed to reduce EAT volume, including exercise interventions, dietary interventions, bariatric surgery, and pharmaceutical interventions^129^, ^130^, ^131^, ^132^, ^133^, ^134^ (Figure 3). There have been a number of well‐designed tables summarizing related clinical studies in recent systematic reviews.^134^, ^135^, ^136^, ^137^, ^138^, ^139^, ^140^, ^141^, ^142^

### 6.1 Exercise and dietary interventions

Numerous studies have investigated the impact of exercise on EAT amount.^135^, ^136^, ^137^ A variety of exercise training protocols were evaluated in these studies, including aerobic exercise, high‐intensity exercise, and resistance exercise, and the length of programs varies from 3 to 24 weeks.^133^, ^138^, ^143^, ^144^, ^145^, ^146^, ^147^, ^148^, ^149^ Most of these studies have reached relatively consistent conclusions that exercise can significantly reduce EAT amount. Some of the studies also found that exercise training can improve metabolic function in patients with MetS by decreasing BMI, weight, and waist circumference.^145^, ^150^ However, no confirmative conclusions can be made yet as there are also similar studies that yielded negative results.^137^ Furthermore, some studies have suggested that exercise also improved inflammatory profiles (i.e., reduced TNF‐α and CRP) in patients with MetS; however, further validation is still needed.^149^, ^151^ Taken together, it has clearly been demonstrated that exercise training is an effective method of reducing EAT amount. However, it should be noted that there is currently no direct evidence to prove that reduced EAT mass mediates the cardioprotective effects of exercise. In addition, no studies have explored the effect of exercise on the radiomics phenotype of EAT, which might be a promising future research direction that can bring us new insights in EAT.

Dietary intervention is another strategy in reducing EAT amount. Some studies restricted caloric intake at a relatively low level (range: 450−1000 kcal/day).^152^, ^153^, ^154^ Others took a more moderate strategy, restricting caloric intake at approximately 1500 kcal/day, equivalent to about 75% of the daily diet.^155^, ^156^ All the studies reported a significant reduction in EAT volume or pericardial adipose tissue volume.

### 6.2 Bariatric surgery

Multiple studies have explored the effect of bariatric surgery, including sleeve gastrectomy and Roux‐en‐Y gastric bypass, on reducing EAT amount.^131^, ^139^, ^150^, ^157^, ^158^, ^159^, ^160^, ^161^, ^162^, ^163^, ^164^, ^165^, ^166^ Most of them demonstrated a significant association between bariatric surgery and reduction in EAT amount; only one of these studies has reported a negative result (nonsignificant change in EAT amount), which might be due to a relatively shorter follow‐up duration (3 months).^162^ According to a meta‐analysis, sleeve gastrectomy is more effective in decreasing EAT than Roux‐en‐Y gastric bypass.^139^ Wu et al.^150^ compared the effects of bariatric surgery and exercise training on reducing EAT amount and found that bariatric surgery leads to more pronounced effects in decreasing excessive fat deposits (including abdominal VAT, abdominal SAT, and PAT) compared with exercise training; however, there was no significant difference in the effect of reducing EAT between the two groups.

### 6.3 Pharmaceutical interventions

Numerous classes of drugs have been used to target EAT amount; antidiabetic and lipid‐lowering drugs are the most studied among them.^129^, ^140^, ^141^, ^142^, ^167^, ^168^, ^169^, ^170^, ^171^, ^172^, ^173^, ^174^, ^175^

#### 6.3.1 Sodium‐glucose cotransporter‐2 inhibitors

Sodium‐glucose cotransporter‐2 (SGLT‑2) inhibitors are a new type of antidiabetic drug that significantly reduce cardiovascular incidence and mortality in patients with T2DM.^176^, ^177^, ^178^ Furthermore, existing evidence has shown that SGLT‑2 inhibitors can significantly reduce body weight and VAT.^179^, ^180^ Thus, several studies further explored their effect on EAT, which is considered a special part of VAT. Currently, there are relatively few studies investigating the effect of SGLT‐2 inhibitors in reducing EAT, and most of relevant studies were carried out in relatively small patient groups with T2DM. Iacobellis et al. ^234^ showed that a 24‐week administration of dapagliflozin significantly reduced EAT thickness in patients with T2DM. Similarly, several other studies demonstrated that treatment with SGLT‐2 inhibitors (including dapagliflozin and canagliflozin) decreased EAT volume measured by CT in patients with T2DM.^129^, ^169^ However, there are also studies with contrary findings, for instance, the RCT by Gaborit et al.^181^ showed that empagliflozin had no significant effect on reducing EAT amount.^142^ For summary, at present, it remains controversial whether SGLT‐2 inhibitors are capable of reducing EAT amount, and subsequent studies with larger sample sizes are needed.

#### 6.3.2 Glucagon‐like peptide‐1 analogues

Some studies have investigated the effect of glucagon‐like peptide‐1 (GLP‐1) analogues, another new emerging antidiabetic drug, on reducing EAT amount.^132^, ^182^, ^183^, ^184^ GLP‐1 analogues have been proved to be associated with decreased body weight and reduced VAT, suggesting that GLP‐1 might be effective in reducing EAT amount as well.^185^, ^186^, ^187^, ^188^ Moreover, it has been reported that the GLP‐1 receptor is expressed in EAT,^175^, ^189^ further providing theoretical support for GLP‐1 analogue‐based pharmaceutical intervention on EAT. As expected, evidence from RCTs showed that treatment with GLP‐1 analogues can significantly reduce EAT amount in patients with T2DM.^167^ GLP‐1 analogues have been demonstrated to reduce major adverse cardiovascular event in patients with T2DM. As mentioned above, in diabetic state, EAT expands in volume and remodels to a proinflammatory phenotype, which is considered deleterious to surrounding tissue. Thus, it is speculated that reducing EAT amount might be a possible mechanism of the cardioprotective effect of GLP‐1.

#### 6.3.3 Statins

Statins are the most commonly used drugs to control dyslipidemia and reduce the risk of subsequent cardiac events.^174^, ^190^ Statins are also effective in reducing EAT amount ^174^,^191^ as well as in ameliorating systematic inflammation in patients with dyslipidemia. Animal studies proved that statin treatment ameliorated inflammation levels in adipose tissue of obese mice and rats with MetS, indicating that statins may be also effective in alleviating inflammation of EAT in the human body.^192^, ^193^ Moreover, several RCTs have demonstrated that statins effectively lowered the prevalence and incidence of AF as well as the risk of AF recurrence.^194^, ^195^, ^196^ Since EAT inflammation is associated with AF, it is speculated that the efficacy of statins in preventing AF may be partially achieved by reducing inflammation of EAT. However, further efforts are warranted to arrive at conclusive evidence.

In summary, all the four interventions, including exercise, diet, bariatric surgery, or pharmaceutical interventions, have proved effective in reducing the amount of EAT.^134^ However, to date, none of the available strategies can specifically target EAT, and it is difficult to conclude that the cardioprotective effects of these interventions are mediated by reduced EAT amount. Thus, targeting EAT as a therapeutic strategy has a long way to go. Further studies are warranted to confirm the independent effects of modulated EAT on CVDs.

## 7 CONCLUSION

**FIGURE 4:**
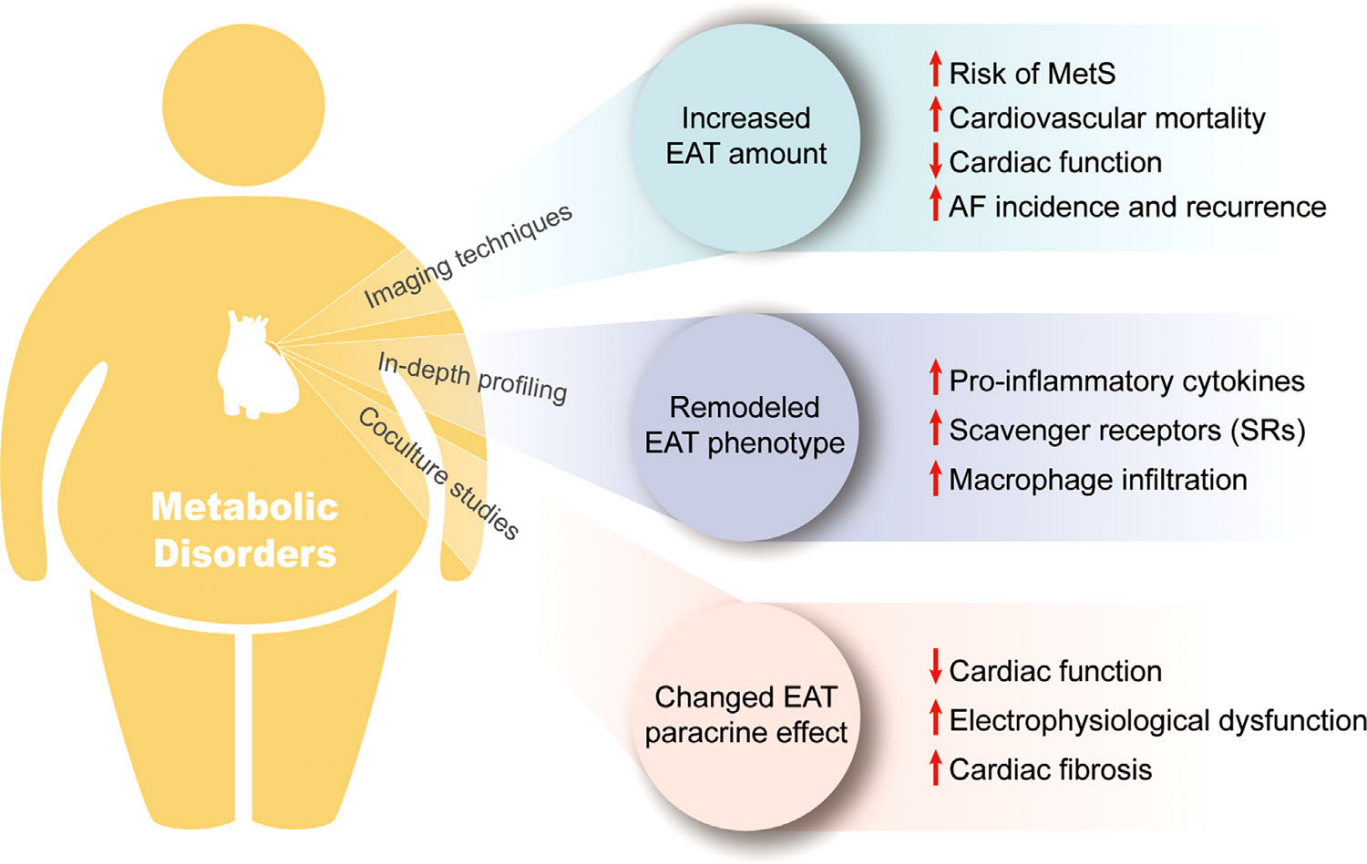
Association between epicardial adipose tissue (EAT) and metabolic disorders. EAT amount measured by imaging techniques is significantly higher in patients with metabolic disorders. An increased EAT volume is associated with a higher risk of developing metabolic syndrome, cardiovascular mortality, atrial fibrillation (AF) incidence, and reduced cardiac function. Further, in‐depth profiling studies suggest that EAT is remodeled into a more proinflammatory phenotype in patients with metabolic disorders. Finally, coculture studies explored the paracrine effect of EAT and provided preliminary evidence that EAT might be a link between metabolic disorders and CVDs.

This review summarizes the latest progress in the field of EAT research, specifically its association with cardiometabolic risk factors and CVDs. In a sequential order, studies based on imaging techniques provided the first insight into the association between the amount of EAT and multiple CVDs, which spurred further investigation of EAT. Further, in‐depth profiling techniques, such as transcriptomics and proteomics, made it possible to obtain a holistic picture of the EAT phenotype at the transcript or protein level, enabling further analysis of the association between the EAT phenotype, cardiometabolic status, and CVDs. These studies also suggested possible key signaling pathways related to metabolic disorders and CVDs. However, neither of the former two types of studies could establish a causal relationship between the properties of EAT and CVDs, let alone the underlying mechanisms. In order to address these limitations, in vitro coculture studies were designed and served as a complement in the field of EAT research, providing ideas for studying the cause–effect link between EAT and CVDs, together with the underlying mechanisms (Figure 4). Finally, the most recent steps in this field were taken to target EAT as a therapeutic strategy for CVDs; however, these efforts are still at an immature stage and require further studies.

The main limitation of this paper lies in the inherent drawbacks of a narrative review. Since this review aims to summarize the role of EAT in CVDs, especially in patients with metabolic disease, as well as to provide an overview of the methodological constructs of the studies in the field of EAT research, multiple categories of studies are involved, including clinical and basic studies; thus, a systematic review does not serve the purpose of this review. In this respect, it should be noted that the level of evidence of the studies included in this review is not consistent. In the sixth part (targeting EAT as a therapeutic strategy), most studies included are RCTs and corresponding meta‐analyses, which are of the highest level of evidence. The third part (clues from studies based on imaging techniques) mainly included cross sectional studies, with relevant lower levels of evidence. This issue is more prominent in the fourth and fifth parts, which involve studies exploring the function mechanisms of EAT. Since research into EAT is still in its early stages, and the specific mechanisms by which EAT functions in CVDs remain largely unknown, many conclusions still need further validation, which has been noted in the main text. For instance, the physiological functions of EAT still lack sufficient direct evidence. Another example is that EAT is found to be more proinflammatory in patients with metabolic disorders, suggesting that EAT may serve as a local mediator of the deleterious effects of cardiometabolic conditions. However, there is also no direct evidence proving that EAT inflammation do promotes CVD progression, such as atherosclerosis or AF. Furthermore, limited by the lack of specific and practical interventions targeting EAT, in vitro studies are the major modality that can directly draw a causal relationship between EAT and CVDs. However, it is obvious that the in vivo environment significantly differs from in vitro conditions, which leads to a low grade of evidence of in vitro studies. For example, the extent to which the secretory products of EAT, such as CM or EVs, produced under in vitro conditions are similar to those produced under in vivo conditions remains to be evaluated. The conclusions of such studies will turn invalid if the in vitro secretory products of EAT are far from being representative of the in vivo secretory products. Therefore, a clear gap exists between in vitro studies and in vivo pathologic processes. Nevertheless, in vitro studies are valuable as they provide preliminary evidence of the casual relationship between remodeled EAT and the progression of CVDs; therefore, they are included in this review.

The past two decades have witnessed a promising advance in EAT‐related research; both clinical and basic studies have demonstrated the important role of EAT in CVDs, indicating the great potential of EAT as a novel target for prediction and treatment of CVDs in the near future. Nonetheless, there is still great scope for further investigations. First, there is a great need for a feasible intervention that can specifically target EAT, such as a method that specifically reduces the inflammatory level of EAT, which will pave the way for clinical studies to confirm the casual relationship between EAT and CVDs and potentially become a new therapy. Second, studies investigating the molecular mechanisms by which EAT exerts its effects are still in its infancy. The progress of mechanism research is meaningful as it can provide guidance for developing therapies targeting EAT. On the basis of the first two points, EAT may serve as a new drug delivery platform by specifically overexpressing a cardioprotective adipokine, which will function in a paracrine manner. Third, surgical intervention of EAT during cardiac operation may be worth considering in the future if we achieve an in‐depth understanding of EAT. Finally, the application of radiomics in EAT‐related research is a novel and promising research direction that may bring a brand‐new perspective in image‐technique‐based studies.

## AUTHOR CONTRIBUTION

All authors have read and approved the final manuscript. W. Y., Y. S., and Y. J. S. conceived the framework of the manuscript. Y. J. S. and M. D. drafted the manuscript and created the figures. Y. Z. T. provided important guidance for the review and revised the manuscript. W. J. S., W. Y. Z., B. Z., and J. C. assisted in literature searching and summary. L. L. F., L. S., M. Z., and Y. Y. L. assisted in polishing the figures. Y. J. S., Y. Z. T., and M. D. contributed equally to this article. W. Y. and Y. S. supervised the work, approved the final manuscript, and in charge of the correspondence.

## CONFLICT OF INTEREST STATEMENT

The authors declare no conflict of interest.

## ETHICS STATEMENT

This review does not require an ethical statement.

## Data Availability

Not applicable.
